# Acute drop of platelets in metastatic colon cancer

**DOI:** 10.1002/ccr3.1210

**Published:** 2017-09-27

**Authors:** Ahmed‐Tarig Ahmed, Shweta Gupta

**Affiliations:** ^1^ Division of Hematology‐Oncology John H. Stroger Hospital of Cook County 1900 W. Polk St, Suite #750 Chicago 60612 Illinois

**Keywords:** Drug‐related thrombocytopenia, immune induced, oxaliplatin, thrombocytopenia

## Abstract

Oxaliplatin is a platinum commonly used in the treatment of metastatic colon cancer. It can cause thrombocytopenia through different mechanism. Sudden isolated drop in platelets should raise the concern for oxaliplatin immune‐induced thrombocytopenia and abrupt discontinuation of the drug. Patients should not be rechallenged with oxaliplatin once diagnosis of OIIT is confirmed.

## Case Presentation

Forty three‐year‐old white woman was diagnosed with metastatic, K‐RAS wild type, colon cancer in July 2013 with liver metastases. She underwent nine cycles of mFOLFOX6 with Bevacizumab. Oxaliplatin was then discontinued due to grade II neuropathy. She was then treated with 5‐FU and Bevacizumab. She then progressed and subsequently received ten cycles of FOLFIRI and Bevacizumab. Her disease progressed again and then switched to FOLFIRI/Cetuximab. Again, her disease progressed so she was started on FOLFOX–Bevacizumab.

Patient tolerated the first three cycles well and had no neuropathy. When she presented for cycle 4, she was found to have severe thrombocytopenia of 6000 k/*μ*L. Peripheral smear revealed large platelets and no schistocytes. She was not prescribed any new medications. Using the Naranjo adverse drug reaction probability scale, the diagnosis of oxaliplatin‐induced thrombocytopenia was probable. She was treated with steroids for suspected ITP. Count recovered above 100 k/*μ*L within 1 week.

**Figure 1:**
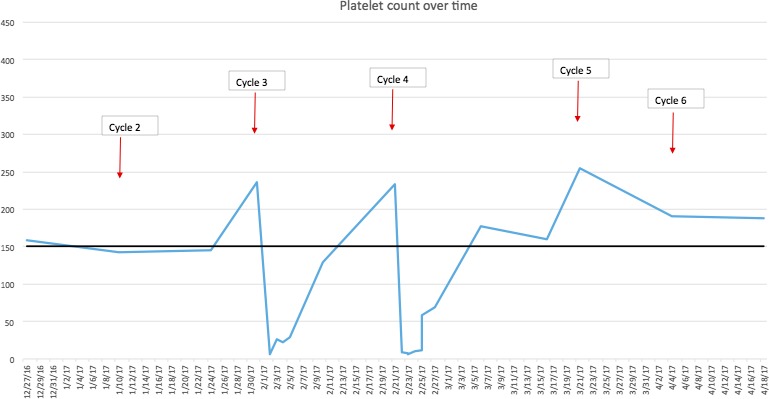
Platelet count over time.

She was rechallenged with mFOLFOX6 with Bevacizumab when her platelet count recovered. The next day her count dropped from 234 k/*μ*L to 9 k/*μ*L when she presented with epistaxis. At this point, her Naranjo score was nine, suggesting oxaliplatin as a definitive cause of her thrombocytopenia. She was treated with IV immunoglobulins, steroids and her counts recovered. Oxaliplatin was dropped with her subsequent cycles, and platelet count remained within normal range (Fig. 1).

## Discussion

Oxaliplatin is a third‐generation platinum, which exerts its effect mostly through DNA damage. It is commonly used in the treatment of colon cancer among other malignancies. The addition of oxaliplatin to 5‐fluorouracil (5‐FU) and folinic acid lead to improved survival in the metastatic disease and reduces the risk of recurrence in stage III CRC. In these studies, the incidence of thrombocytopenia of all grade was up to 77%, which is significantly higher than the rate observed when 5‐FU and folinic acid are used without oxaliplatin 2. Grade 3–4 thrombocytopenia is rare, and it represents about 3–4% of patients exposed to oxaliplatin, and tends to increase with repeated exposures (Table 1).

**Table 1:** Naranjo Score

Question	Yes	No	Do not know	Score 1	Score 2
Is there published evidence that the reaction has been described?	+1	0	0		
Did the side effect develop after drug administration?	+2	−1	0		
Did the side effect improve/resolve with discontinuation of the drug?	+1	0	0		
Did the reaction reappear when patient rechallenged?	+2	−1	0		
Is there an alternative etiology?	−1	+2	0		
Did the side effect recur when a placebo given?	−1	+1	0		
Was the drug level detected or supratherapeutic?	+1	0	0		
Was the reaction severe with dose escalation or less with dose reduction?	+1	0	0		
Any previous similar reaction to the same drug?	+1	0	0		
Was the side effect confirmed by a laboratory test?	+1	0	0		
Total score				5	9

Naranjo adverse drug reaction probability scale 1.

The most common cause of oxaliplatin‐induced thrombocytopenia is suppression of the bone marrow, such as other platinums. This usually occurs after about 1 week following chemotherapy administration and usually recovers by the next cycle of chemotherapy.

Oxaliplatin immune‐mediated thrombocytopenia is another mechanism of oxaliplatin‐induced immune thrombocytopenia (OIIT). The true incidence of it is not known. In the presence of oxaliplatin, antiplatelet antibodies have higher affinity to platelets antigens, which results in platelet destruction and thrombocytopenia 3. Oxaliplatin‐induced antibodies against glycoprotein IIb/IIIa complex are most commonly involved in immune‐mediated thrombocytopenia 4, but other antibodies against other platelet surface glycoproteins, such as GP Ia/IIa and GP Ib/IX, have been identified 5, 6. These antibodies react in the presence of oxaliplatin but neither react in the presence of carboplatin, 5‐FU or folinic acid, suggesting specificity for oxaliplatin 4.

The usual presentation of oxaliplatin‐induced immune thrombocytopenia is rapid drop in platelets with a nadir as low as 2 × 10^9^/L. Females with advanced‐stage CRC with prior oxaliplatin exposure are commonly affected, and it tends to occur after multiple exposures to oxaliplatin. Thrombocytopenia can also be preceded by hypersensitivity reactions, that is, skin rash, pruritus, chills, and bronchospasm 7.

Identifying the antibodies that interact with platelets in the presence of the sensitizing drug is possible, but the required testing is not widely available and generally performed at a reference laboratory. When the test is not available, high index of suspicion, clinical judgment, and use of adverse drug reaction probability scale such as Naranjo scale can be helpful for diagnosis.

Idiopathic immune thrombocytopenic cannot initially be differentiated from OIIT, and so the use of steroids and intravenous immunoglobulins is justifiable. Platelets recovery is usually within few days and complete after cessation of the drug. Patients should not receive oxaliplatin again in the future as drug sensitivity usually persists indefinitely 3.

Another mechanism of thrombocytopenia caused by oxaliplatin can occur due to sinusoidal injury, leading to fibrosis and veno‐occlusive disease resulting in portal hypertension and ultimately splenomegaly with associated thrombocytopenia 8.

The development of splenomegaly has been directly correlated with the cumulative amount of oxaliplatin administered. The reported rate of splenomegaly with oxaliplatin use is up to 86% which correlate with the degree of thrombocytopenia 8. It tends to occur after a median of twleve cycles of oxaliplatin has been administered 8. The drop in platelets is not acute such as OIIT, and it presents as a moderate prolonged thrombocytopenia with a mean count of 81 × 10^9^/L, and it rarely causes bleeding complications 9, 10. Bone marrow aspiration is not indicated, but, if carried out, it will likely reveal normal hematopoiesis 9. In the absence of splenomegaly, other causes of thrombocytopenia should be considered, including bone marrow suppression and drug‐induced immune thrombocytopenia.

Discontinuation of oxaliplatin is the main stay of treatment in this setting. Platelet recovery is usually slow and counts reach baseline levels in 2–3 years after oxaliplatin cessation 10.

In our patient presented in this report, the acute drop in platelets and rapid count recovery within days suggests oxaliplatin‐induced immune thrombocytopenia, which was discontinued in the subsequent cycles, and her count remained within normal range.

## Conclusion

Oxaliplatin can cause thrombocytopenia through different mechanisms including bone marrow suppression, immune‐induced thrombocytopenia, and splenic sequestration. OIIT should be considered when there is isolated acute drop in platelet count. Patients should not be rechallenged with oxaliplatin once diagnosis of OIIT is confirmed.

## Authorship

ATA and SG: have developed the concept of the case report and drafted the manuscript. ATA: wrote the case and revised the final manuscript.

## Conflict of Interest

None declared.
